# 

**Published:** 1887-04

**Authors:** 


					APRIL, 1887.
THE
AMERICAN JOURNAL
miO-OF^
DENTAL SCIENCE.
EDITED BY
F. J. S. GORGAS, M. D., D. D. S.
t?3;
o
S
Pxj
EE
cn
i?
PC
CU
GO
CN?
cn
O
t~d
25
2;
c=J
Uoii. so
THIRD SERIES.
ft O. 12.
BALTIMORE:
SNOWDEN & COWMAN.
LONDON:
TRUBNER & CO., 60 PATERNOSTER ROW.
:PM?BLE IN ADVANCE.:

				

